# Physical working conditions as covered in European monitoring questionnaires

**DOI:** 10.1186/s12889-017-4465-7

**Published:** 2017-06-05

**Authors:** Tore Tynes, Cecilie Aagestad, Sannie Vester Thorsen, Lars Louis Andersen, Merja Perkio-Makela, Francisco Javier Pinilla García, Luz Galiana Blanco, Greet Vermeylen, Agnes Parent-Thirion, Wendela Hooftman, Irene Houtman, Falk Liebers, Hermann Burr, Maren Formazin

**Affiliations:** 1 Statens arbeidsmiljøinstitutt (National Institute of Occupational Health), Gydas vei 8, Majorstuen, Oslo, Norway; 2 Det Nationale Forskningscenter for Arbejdsmiljø (National Research Center for the Working Environment), Lersø Parkallé 105, 2100 København Ø, Denmark; 3 Työterveyslaitos (Finnish Institute of Occupational Health), Topeliuksenkatu 41 b, 00250 Helsinki, Finland; 4 Instituto Nacional de Seguridad e Higiene en el Trabajo (National Institute of Safety and Hygiene at Work), Calle de Torrelaguna 73, 28027 Madrid, Spain; 5 European Foundation for the Improvement of Living and Working Conditions (Eurofound), Wyattville Road, Loughlinstown, Dublin, 18 Ireland; 6 TNO, Postbus 3005, 2301 DA Leiden, Netherlands; 7 Bundesanstalt für Arbeitsschutz und Arbeitsmedizin (Federal Institute for Occupational Safety & Health), Department “Work & Health”, Nöldnerstraße 40-42, 10317 Berlin, Germany

**Keywords:** Physical workloads, Mechanical exposures, Monitoring, Surveillance, European dimension

## Abstract

**Background:**

The prevalence of workers with demanding physical working conditions in the European work force remains high, and occupational physical exposures are considered important risk factors for musculoskeletal disorders (MSD), a major burden for both workers and society. Exposures to physical workloads are therefore part of the European nationwide surveys to monitor working conditions and health. An interesting question is to what extent the same domains, dimensions and items referring to the physical workloads are covered in the surveys. The purpose of this paper is to determine 1) which domains and dimensions of the physical workloads are monitored in surveys at the national level and the EU level and 2) the degree of European consensus among these surveys regarding coverage of individual domains and dimensions.

**Method:**

Items on physical workloads used in one European wide/Spanish and five other European nationwide work environment surveys were classified into the domains and dimensions they cover, using a taxonomy agreed upon among all participating partners.

**Results:**

The taxonomy reveals that there is a modest overlap between the domains covered in the surveys, but when considering dimensions, the results indicate a lower agreement. The phrasing of items and answering categories differs between the surveys. Among the domains, the three domains covered by all surveys are “lifting, holding & carrying of loads/pushing & pulling of loads”, “awkward body postures” and “vibrations”. The three domains covered less well, that is only by three surveys or less, are “physical work effort”, “working sitting”, and “mixed exposure”.

**Conclusions:**

This is the fırst thorough overview to evaluate the coverage of domains and dimensions of self-reported physical workloads in a selection of European nationwide surveys. We hope the overview will provide input to the revisions and updates of the individual countries’ surveys in order to enhance coverage of relevant domains and dimensions in all surveys and to increase the informational value of the surveys.

**Electronic supplementary material:**

The online version of this article (doi:10.1186/s12889-017-4465-7) contains supplementary material, which is available to authorized users.

## Background

Monitoring working conditions using national and / or EU wide surveys is an important method to obtain data necessary for policy decisions regarding work and health. The monitors have two important aims: (i) to study status and trends over time within the workforce, and (ii) to study differences between groups defined by e.g. industry, occupation, age and gender. National as well as European politicians and social partners use – among other aspects – the results from these population-wide surveys as a foundation for their decisions regarding e.g. the necessity of regulation or allocation of resources [1, 2].

Working conditions include a broad spectrum of risk factors like chemical, biological and other physical exposures (noise, extreme temperatures, etc.), the risk of injuries, a wide range of psychosocial working conditions, e.g. quality of leadership and recognition, and also ergonomic risk factors or physical workloads, e.g. lifting of heavy loads and awkward body postures. Whereas the dimensional comparability of psychosocial working conditions as covered in European monitoring questionnaires has already been assessed [3], no overview or comprehensive comparison exists for physical working conditions. In the context of this paper, physical working conditions are defined as occupational exposures of the body with possible relevance for musculoskeletal health. Thus, physical working conditions involve lifting, holding and carrying loads, pushing and pulling loads, repetitive manual work processes (i.e. involving small muscle groups of the hand-arm system), whole-body forces (i.e. involving larger muscle groups such as the extremities and lower back), awkward body postures (i.e. static muscle tension of – or passive pressure on – neck, torso or the extremities) and dynamic body movements (i.e. dynamic use of larger muscle groups, e.g. climbing, crawling, walking stairs), as well as mechanical exposures like vibrations. Similar dimensions of physical labour were distinguished both by the Norwegian labour inspectorates’ report [4] based on work quantifying variation in physical load [5] and in the German research project “MEGAPHYS” (BAuA project no. F 2333)^1^ which – among other aspects – aims at further developing the key indicator methods as a screening method for risk assessment of physical workload [6]. There are two reasons for monitoring physical workloads: First, they have been shown to predict workers’ health and labour market participation; second, they are prevalent in the European work force. Both aspects are described in the following.

### Physical workloads and health

Physical workloads have been found to be related to both musculoskeletal complaints [7, 8] and labour market participation [9–11].

They can cause specific serious conditions (diseases) [12–19]. Some of these conditions are listed as occupational diseases in Europe [20]. Regarding labour market participation, physical workloads have been found to predict sickness absence [9, 21–31] and disability pensioning [32–41]. The physical exposures related to sickness absence are to some extent the same as those related to disability pensioning as well [32–41]. One can hence summarize that the plethora of results on the association of workers’ physical workloads and their health and labour market participation underlines why it is important to measure them.

### Prevalence of physical exposures

In spite of attention among both the scientific and the political community, the prevalence of workers with demanding physical workloads in the European work force remains high. In 2015, the fraction of workers in the EU reporting – in at least ¼ of working hours – tiring or painful positions was 43%, carrying heavy loads 32% and vibrations 20% (The European Foundation for the Improvement of Living and Working Conditions (Eurofound) [42]). Similarly, high levels of exposure have been reported based on national monitoring data [43–46].

Altogether, this shows that 1) there is scientific evidence on the association of physical work exposures and health effects, and 2) a high number of workers is exposed to these conditions. In spite of this, a comparison of quality and quantity of exposures across EU countries is difficult because currently, no consensus exists regarding a standardized approach to be used in surveys to measure physical work exposure. The European Working Conditions Survey (EWCS), carried out in 35 European nations and covering 44,000 workers in 2015, and containing five physical working condition items, is at present our best starting point for comparisons [42].

### Aim

In this paper, our aim is to establish an overview of physical workloads as measured in European monitoring instruments. Based on this overview, we will examine 1) which domains and dimensions of the physical workloads are monitored in surveys at the national level and the EU level and 2) the degree of European consensus among these surveys regarding coverage of individual domains and dimensions.

## Methods

### Data

In 2009, six member countries of the “Partnership for European Research in Occupational Safety and Health (PEROSH)”, a joint collaboration of European institutes on research and development in occupational safety and health, formed a project group and in 2014 published an article on the comparability of psychosocial working conditions measured in European monitoring questionnaires [3]. Inspired by this work, the group decided to continue its cooperation in 2015, now with a focus on physical workloads measured in the national surveys. The items from the following surveys were included in the comparative review: National Working Condition Survey (NEA) in the Netherlands, Finnish National work and health survey (FNWHS), Norway - Survey of living conditions - Working environment (LKU, Statistics Norway), Work Environment and Health in Denmark (NRCWE), German Labour Force Survey (BIBB/ BAuA) and the European Working Conditions Survey. Spain uses the EWCS questionnaire in an enlarged sample to measure status and trends of working conditions, hence, the items measuring physical exposures in Spain and in EWCS are therefore identical. All included surveys are repeated periodically. For each survey, the latest wave with an English translation was selected. Professional translators in each country translated the items from their original language into English. There are two exceptions: in Germany, professional translators only translated the older version of the survey from 2006, and two English-speaking scientists from the field of occupational safety and health translated the items that were new in the latest survey; in Denmark, only a preliminary translation of the questionnaire exists as of yet. In an additional file (see Additional file 1: Description of the EU-wide and the national surveys included in our paper), we have provided a more detailed description of the surveys, also including a reference list of epidemiological papers and presentations based on the surveys.

### Analysis

After consulting European experts on physical exposures at work and joint discussions, the cooperating partners agreed on a taxonomy to classify the physical working condition items of all questionnaires. This taxonomy and the sorting are based on consensus among the partners and comprises nine domains (Table 1): (i) lifting, holding & carrying of loads /pushing & pulling of loads, (ii) manual work processes /repetitive hand-arm movements, (iii) working standing/walking, (iv) working sitting, (v) awkward body postures, (vi) physical work effort, (vii) vibrations, (viii) work with computer, (ix) mixed exposure. For six of these domains, a further distinction into two to six dimensions was required to account for the specificity of the aspects covered, leading to a total of 22 dimensions. All items referring to physical workload were evaluated and sorted into one of these 22 dimensions. This assignment was done independently per country by the researchers for the respective countries (TT and CCA for Norway, SVT and LLA for Denmark, MPM for Finland, JVPG and LGB for Spain, AV and AGP for Europe, WH and IH for the Netherlands, HB and MF for Germany) and cross-checked by the other researchers. If items were assigned to different categories, the assignments were discussed and decided by consensus. In cases where an item referred to a combination of working conditions, not allowing for a clear classification into one dimension, this item was placed in the domain “mixed exposures” (Table 1).

**Table 1:** Physical working conditions as covered in the surveys – number of surveys for each domain and dimension

Domain	No. of surveys covering	Dimensions	No. of surveys covering	DK	NO	FI	NL	GER	ES/EU
Lifting, holding & carrying of loads /pushing & pulling of loads	6								
		Lifting, holding & carrying of loads	4	√	√	√	-	√	-
		Pushing and pulling of loads	1	√	-	-	-	-	-
		Mixture of both	2	-	-	-	√	-	√
Manual work processes /repetitive hand-arm movements	5								
		Repetitive movements	5	√	√	√	√	-	√
Working standing / walking	5								
		Working standing only	3	-	√	-	-	√	√
		Working walking only	1	-	√	-	-	-	-
		Mixture of standing and walking	2	√		√	-	-	-
Working sitting	3								
		Working sitting	3	√	√	√	-	-	-
Awkward body postures	6								
		Work at or above shoulder height	3	√	√	√	-	-	-
		Working with back in an awkward position	3	√	√#	√	-	-	-
		Working squatting or kneeling	2	√	√	-	-	-	-
		Working with head bent forward	1	-	√	-	-	-	-
		Working in specified awkward body postures (above shoulder height, bended back, kneeling)	1	-	-	-	-	√	-
		Work in uncomfortable and tiring body postures	2	-	-	-	√	-	√
Physical work effort	3								
		Strenuous work	3	√	√	√	-	-	-
		Strenuous lifting	1	√	-	-	-	-	-
Vibrations	6								
		Vibrations unspecified	4	√	-	√	-	√	√
		Arm/hand vibrations	2	-	√	-	√	-	-
		Whole body vibrations	1	-	√	-	-	-	-
Work with computer	4								
		Work with computer/laptop/smartphone	4	-	√	√	-	√	√
Mixed exposures	2								
		Strong gripping and turning movements of the hand	1	-	-	√	-	-	-
		Manual work that requires great dexterity, fast sequences of movements or greater strength	1	-	-	-	-	√	-
Number of domains per survey				7	8	9	4	6	6
Number of dimensions per survey				11	13	10	4	6	6

√ = item available in this survey; − = item not available in this survey. *DK* Denmark, *NO* Norway, *FI* Finland, *NL* Netherlands, *DE* Germany, *ES* Spain, *EU* Europe. Spain uses the EWCS questionnaire in an enlarged sample. Hence, the items measuring physical exposures in Spain and in EWCS are identical and therefore presented in one column. # Question asked in NO: “Do you work in positions where you are leaning forward without supporting yourself on your hands or arms?” With two follow up questions; (i) “with back twisted?”; and (ii) “lifting more than 10 k?”

## Results

Table 1 gives an overview of the domains and dimensions of physical work demands or exposures used in the six surveys (EWCS and Spain combined in one column). We give a more thorough overview of all dimensions and the specific formulation of all items in Additional file 2: Overview of all dimensions and items assessing physical workloads in the six surveys.

Five of the six surveys cover six or more of the nine domains. The Finnish survey covers all nine domains, the Norwegian eight, the Danish seven, the German and the European/Spanish cover six each, and the Dutch survey covers only four domains. The results indicate a modest degree of consensus among the surveys with regard to the coverage of domains. The three domains covered by all surveys are “lifting, holding & carrying of loads/pushing & pulling of loads”, “awkward body postures” and “vibrations”. The three domains covered less well, that is only by three surveys or less, are “working sitting”, “physical work effort” and “mixed exposure”.

When considering dimensions in the domains, the results indicate lower agreement. None of the 22 dimensions is covered by all six surveys.

Only one out of 22 dimensions is covered by five of the six surveys, namely “repetitive movements”, the three dimensions” lifting, holding & carrying of loads”,” vibrations unspecified” and “work with computer/laptop/smartphone” are covered by four surveys. However, whole body vibration is specifically only addressed in one survey. There are five dimensions covered by three surveys, while the remaining dimensions are covered by one or two surveys only. Due to the specificity of items, the two dimensions in the domain “mixed exposures” are each covered by one survey only.

The Norwegian survey covers 13 out of 22 dimensions, indicating a fairly good coverage in the assessment of physical workloads. To a somewhat smaller extent, this also holds true for the Danish survey with 11 dimensions and the Finnish survey with 10 dimensions. The other three surveys cover only one dimension per domain.

Even though there is some similarity across countries in which dimensions are covered by the individual surveys, one can find many differences in the wording of items as well as answering schemes assessing the different dimensions. For the former, one example is the assessment of “strenuous work”: In Denmark and Finland, workers are asked whether their work is strenuous, whereas Norwegian workers are asked if their work involves so much effort that it causes them to breathe more rapidly. Another example is “work with computer/laptop/smartphone” where the focus in Norway is on work with keyboard and mouse and work in front of a computer screen, whereas the Finnish survey focuses on whether the computer is a laptop or not, and if the workplace has a separate keyboard and screen. In Germany, an item about the frequency of internet use and processing e-mails follows the general item on work with computers.

Similarly, answering schemes between countries partly differ with regard to both the number of categories and their labelling. Several countries, e.g. Denmark, Norway, Spain/EWCS, use fractions of the day (e.g. “approximately 3/4 of the time, approximately 1/2 of the time, approximately 1/4 of the time”) as answering categories, whereas the Netherlands and Germany use answering categories such as “yes, regularly”/“frequently” and “yes, sometimes”/“sometimes”. The German survey also includes the category “rarely” to assess the frequency or intensity level of the exposure.

## Discussion

This paper aimed at answering two questions regarding monitoring of working conditions in Europe. First, which domains and dimensions of the physical working environment are monitored in the European wide survey also used in Spain and the five national surveys included in our paper. Second, we assessed the degree of European consensus among these surveys regarding coverage of individual domains and dimensions.

The answer to the first question is that there is a modest overlap between the domains measured in the different surveys. This overlap in domains could indicate that scientific knowledge of potential associations between specific exposures and outcomes has influenced the selection of domains measured in these surveys in the process of development of the national surveys. At the same time, it could indicate that people with hands-on knowledge of work places, e.g. physiologists or labour inspectors with shop floor experience, have independently pointed out these domains. Moreover, we can assume that the same themes are of relevance for the labour inspectorates and the social partners in the different countries.

When dimensions are evaluated, the results are more mixed and the degree of consensus is smaller: the number of dimensions covered across countries varies widely between four and 13 of the total possible 22. This result points to a rather country-specific focus in the selection of dimensions when compiling the instruments.

Even though all monitoring instruments measure the physical work environment, the actual items applied vary considerably between countries. One can hence assume that the choice and formulation of items in each country are to a much stronger degree influenced by national political negotiation processes than by international scientific deduction. Summarizing the similarities and differences across the instruments, one can state that there is a potential for a better harmonization of surveys regarding the coverage of physical workloads across Europe. Whole body vibration is an example of an item where the coverage could be improved.

Which reasons can be given for the different levels of detail in the domains in assessing the physical work environment, e.g. “lifting, holding & carrying of loads /pushing & pulling of loads”? This domain is covered in all countries and the EWCS: Dutch workers are asked a general question on whether the job requires the respondents to apply a lot of force (pushing/lifting/etc.) or to use equipment that requires a lot of force. Respondents in Denmark, Norway, Finland, Germany and Spain/EWCS are asked if they are lifting or lifting/carrying objects. In the first four of these countries, the questionnaire refers to a specified weight, e.g. in Germany, men are asked if they are lifting and carrying more than 20 kg, while the weight specified for women is more than 10 kg. In Norway, there are two items involving heavy lifting, one assessing lifting more than 20 kg and the other more than 10 k. In Finland, the weight categories used are less than 5 k, 5–25 k, or over 25 k; similar categories are used in Denmark.

In order to specify the kind of lifting required, workers in Norway and Spain/EWCS are asked a follow-up item on whether their lifting involves lifting or moving people. In general in Europe, lifting or moving of heavy loads is more prevalent among men than among women, and such heavy lifting is associated with heavy labour in male dominated occupations, while lifting of people is more prevalent in female dominated occupations in the health and social sector [42]. To differentiate between heavy lifting (of loads) and lifting of people seems to be further justified when considering the different prevalence of both aspects across Europe: while workers in the east and southern part of Europe report a high exposure to lifting of heavy loads, workers in northern EU countries to a greater extent report lifting of people [47]. A recent meta-analysis of longitudinal studies assessing the effect of occupational lifting on low back pain (LBP) estimated that lifting loads over 25 kg and lifting at a frequency of over 25 lifts/day will increase the annual incidence of LBP by 4.32% and 3.50%, respectively, compared to the incidence of not being exposed to lifting [48].

ÈWCS and all national surveys, except Germany, specifically cover repetitive hand−/arm movements while this is only indirectly assessed in Germany as a “mixed exposure” through the item referring to “manual work that requires great dexterity, fast sequences of movements or greater strength”. Scientific papers and literature reviews on the development of health-related problems due to this exposure [7, 8, 13, 17–19] support its coverage.

While most of the dimensions measured in the surveys are documented risk factors for specific health complaints, sick leave or disability pension, the domain “working sitting” covered by Denmark, Norway and Finland has recently gained increased interest as a risk factor for health complaints, diseases or all-cause mortality in the literature. A follow-up of all-cause mortality risk based on the Whitehall II study concluded that sitting time was not a risk factor for this outcome [49]. According to the authors, the findings of no association in the study suggest that policy makers should be cautious about recommending sitting reductions without also recommending increases in physical activity. Choi et al. have reported that low physical activity at work is a significant risk factor for obesity in middle- aged male workers, particularly when they worked longer than 40 h per week [50]. A systematic review reported limited evidence to support a positive relationship between occupational sitting and health risks, and the heterogeneity of study designs, measures, and fındings made it diffıcult to draw defınitive conclusions [51].

Occupational musculoskeletal disorders (MSDs) have - in addition to individual factors - been associated both with psychosocial working conditions and - to a stronger degree - with the physical work environment [52, 53]. Today, there is an international near-consensus that MSDs are causally related to occupational physical workloads, such as repetitive and stereotyped motions, forceful exertions, non-neutral postures, vibration, and combinations of these exposures [53]. Taken together, the surveys included in this paper cover a majority of these dimensions (Table 1) and – combined with Additional file 2 of our taxonomy - can be a useful source for ways of assessing these dimensions via new items in upcoming surveys in larger populations.

### Methodological challenges

In musculoskeletal epidemiology, the exposure assessment strategy remains a huge challenge, and there is no perfect instrument for measuring all relevant dimensions of physical loads [54]. The aim in a questionnaire addressing dimensions of physical workloads is to assess frequency, level and duration of certain types of exposure [55, 56]. In epidemiological studies, however, all three dimensions are rarely collected simultaneously. Questionnaires in national surveys are designed to aim at physical workloads in general, and these self-reports can give some insight into the occurrence of tasks and activities, and the approximate proportion of time spent on each of them. At the same time, most estimates of external exposures like the working situation, the actual working method, as well as the triad postures, movements, and exerted forces, are, however, imprecise and inaccurate with too low validity and reliability [55]. Exposure data from self-administered questionnaires on manual materials handling and work postures have been validated in relation to direct measurements and systematic observations [57]. At the dichotomous level, the agreement was “acceptable” for a majority of the variables concerning work postures and for handling of loads weighing >5 kg. No variable, however, showed “acceptable” agreement when the duration or the frequency was quantified in more detail (4- to 6-point scales). A limitation in our study is the fact that it is not exhaustive regarding the existing national working condition surveys in Europe, and access to more studies would have provided a broader picture of the domains and dimensions covered on this topic. A wider scope was not possible due to the limitation to those countries that volunteered to take part in the PEROSH group. In addition, the PEROSH group decided solely to focus on monitoring instruments; hence, questionnaires from other studies with a different scope (e.g. SHARE or SILC) have not been included in the overview.

A limitation regarding the EWCS is the low number of study subjects in each country and a large proportion of non-response [58]. The national surveys may also suffer from low response, and this might limit the generalisability of the results to the targeted populations.

All the survey data included in this paper are collected by self-report, either as telephone or as personal interviews or as self-administered paper questionnaires. Self-report offers the possibility of studying a great number of persons nationwide at a modest cost while allowing the investigation of a large number of variables; it is a feasible method to assess exposures that occur with highly irregular patterns, for example exposures that change seasonally or exposure in the past [59]. Self-report measures are therefore relevant in the context of large population-based surveys that include many job titles, multiple workplaces, and a wide variety of occupational tasks and are designed to monitor general trends of exposure to important determinants of musculoskeletal disorders such as physical load over time [60]. Self-reports may also help to effectively convey relative differences in exposures of heterogeneous populations, but at the same time they are imprecise measures of the absolute levels of the exposure [54]. A review of the validity of self-reported mechanical demands for occupational epidemiologic research of musculoskeletal disorders indicated that among assessments reporting correlations as a measure of validity, studies with a better match between the self-report and the reference method, and studies conducted in more heterogeneous populations tended to report higher correlations [59]. The authors concluded that the use of self-reported mechanical demands for occupational epidemiologic research requires further, better validity testing research, and that the full potential of self-reports in occupational epidemiologic research is still to be discovered. Stock et al., in their systematic review, stated that the validity of self-reports for the assessment of mechanical exposures cannot be appropriately established with the information currently available [60]. Instead, due to the need for practical, questionnaire-based measures for epidemiologic studies, efforts should pertain to improve the design of individual items and their response scales. Stock et al. evaluated self-report items on physical work demands for both reproducibility and validity. Items that performed well included those on duration or presence of sitting and standing posture, the presence of walking, kneeling or squatting postures as well as duration or frequency of hands above shoulders. Manual handling of more than or less than 10 kg, general level of physical effort, presence and duration of whole-body vibration, and duration of the use of visual display terminals also performed well. All dimensions showing good reproducibility and validity in the review by Stock et al. are included in the majority of the surveys in our study. In a recent Norwegian study aiming at determining the criterion validity of the Norwegian survey items on physical load in comparison to objective measurements, the authors concluded that the self-report overestimated the exposure durations; the highest correlations between self-report and measurements were reported for sitting and work with hands above shoulder height [61].

In our taxonomy, work with computer is defined as a domain, but one could also say that work with computer is a mix of exposures, e.g. working sitting down or standing, repetitive work, potential awkward arm−/hand movements and other exposures or demands regarding visual demands or psychosocial influences. A study of computer workers indicated that exposure may also depend upon the usability of the computer software [62]. The authors reported that users frustrated by the software, increased their forces applied to the mouse and increased EMG activity of the wrist extensor muscles. The data also suggest that other factors, such as individual factors, may confound the results: Increased exposures were only observed among those who were critical of the usability of the software.

Another example of mixed exposures is the Norwegian item “Do you work in positions where you are leaning forward without supporting yourself on your hands or arms? If yes, how much of the time do you do this during a normal working day?”, followed by two items” When working in those positions, do you work in those positions with your back twisted?” and” When working like this, do you need to lift anything that weighs more than 10 kg?” The idea with this combination of items is to find workers exposed to both awkward positions and high muscular strain implying large energy expenditure. The number of subjects exposed to this combination of exposures in the national study (in Norway about 9000 economically active take part in the survey) is, however, too small to properly evaluate potential negative muscular health effects of this combination of exposures.

There are still gaps in knowledge concerning the latency of effects of specific physical workloads, the potential of a selection bias in the form of a “healthy worker effect”, and the threshold at which the exposure starts to produce a negative physiological effect. Consequently, prospective longitudinal scientific studies in a large number of countries are needed in order to evaluate which dimensions of the physical work environment are most important for health. The Norwegian survey has a panel design since 2006 as does the Danish survey since 2012 with those that responded in the first round (in 2014: 1/3 of the whole sample). Such a design allows for a longer follow-up. Currently, however, the lack of standardized exposure metrics across surveys limits the ability to compare findings across countries and studies. Therefore, a better harmonization of items and answering categories across studies would be desirable for comparisons across countries. However, changing items in repeated surveys prohibits the analysis of trends over the years. Moreover, the stakeholders involved in these surveys are reluctant to change tools on which agreement has been made. An alternative are studies showing how well the different items and answering categories correlate before joint analysis with other European surveys can be undertaken.

## Conclusions

This is the fırst thorough overview to evaluate the coverage of physical workloads in European nationwide surveys. An important aim of the surveys is to identify the important risk factors at work for diseases and complaints, and poorly characterized risk factors will lead to inadequate assessments. Our study indicates that similar domains of the physical work environment are covered in the majority of the surveys. When considering dimensions, the results are more mixed, and when the same dimensions are covered in the surveys, both the phrasing of items and answering categories differ between them. To what extent the same domains, dimensions and items referring to the physical work environment are covered in the surveys may indicate the degree of consensus among nations regarding the relevant physical exposures for MSD. When revising existing surveys and planning new ones, the information presented in our study may provide some help in choosing the relevant items to include in the national questionnaires. In order to facilitate an improved comparison of status and trends in European nations, a better harmonization of the items covering this topic in the national surveys would be desirable. However, since the national monitors are dependent on national ownership among the social partners, the state and local scientific communities regarding questionnaire content and concrete formulations of questions, the degree to which such measurements can be aligned across countries is limited. Our comparison might also be helpful when developing job exposure matrices (JEM) based on national data for analysis of longitudinal health information via occupational codes in national registries [63]. A sizable proportion of MSDs among exposed workers are preventable, and protective action is therefore both warranted and necessary.

## Additional files

Additional file 1: Description of the EU-wide and the national surveys included in our paper. (PDF 624 kb)

Additional file 2: Overview of all dimensions and items assessing physical workloads in the six surveys. (PDF 65 kb)

## Abbreviations

BAuA: Federal Institute for Occupational Safety and Health (Germany)
BIBB/BAuA: German Labour Force Survey
DE: Germany
DK: Denmark
DWECS: Danish Work Environment Cohort Study
ENCT: Spanish National Working Conditions survey
ES: Spain
EU: Europe
EWCS: European Working Conditions Survey
FI: Finland
FNWHS: Finnish National work and health survey
JEM: Job exposure matrix
LBP: Low back pain
LKU: Norway - Survey of living conditions - Working environment
MSD: Musculoskeletal disorders
NEA: National Working Condition Survey in the Netherlands
NL: Netherlands
NO: Norway
OSH: Occupational safety and health
PEROSH: Partnership for European Research in Occupational Safety and Health

## Footnotes

1
http://www.baua.de/en/Research/Research-Project/f2333.html
