# Supplementary material for: Physical working conditions as covered in European monitoring questionnaires
Source: BMC Public Health. 2017 Jun 5;17:544. doi: 10.1186/s12889-017-4465-7 (PMC5460526; doi:10.1186/s12889-017-4465-7)
Supplement: Supplementary file 1 — Description of the EU-wide and the national surveys included in our paper. (PDF 624 kb) [file 12889_2017_4465_MOESM1_ESM.pdf]

Additional file 1

Description of the EU-wide and the national surveys included in our paper.

All the surveys included in our publication are aiming at collecting timely and comparable cross-sectional and longitudinal multidimensional microdata on income, physical-, mechanical-, biological and psychosocial exposures and data on various health outcomes related to work exposures. Below we present a description of each of the surveys included in the paper. We have also included a reference list including published papers and presentations based on the surveys, addressing physical workload and various health outcomes.

The survey on living conditions - working environment - Norway
was launched by Statistics Norway in 1989 and has been implemented every third year since. Prior to this launch, a group of Nordic researchers compiled the questionnaire with the purpose to make comparative analyses of the work environment possible in the Nordic countries. In 1987, the questionnaire was evaluated through pilot studies in Sweden and Denmark before the final version was introduced. The survey covers many topics where biomechanical exposures is one of them. Before each launch of the survey, stakeholders (social partners, the Labour Inspectorate, the National Institute of occupational and other stakeholders) are invited to suggest revisions of the questionnaire. Eligible respondents for the Norwegian survey are community-living Norwegian residents aged 18–66 years registered in Statistics Norway's population database (BeReg). In 2006, the gross-sample for the survey was expanded from 5,000 subjects to 19,000 subjects and a panel design was introduced. The method of data collection is telephone interviews undertaken by professional interviewers at Statistics Norway. The data files with results from the interviews and statistical files with coded variables, linked information and weights are stored. Anonymized files are also available for researchers through the Norwegian Social Science Data Archives. In the 2013 survey the gross sample was 21,707 subjects, and the number of subjects interviewed were 10,875 (8,302 were in paid work). In recent epidemiological papers evaluating associations with various health outcomes, the predictive validity of the physical load questions has been supported [1-8].

Work Environment and Health in Denmark (WHED)
is a survey of work environment and health launched in 2012 and implemented every second year after. The National Research Center for the Working Environment (NRCWE) is in charge of the study. The objective is to monitor the Danish work environment (the survey is funded by the state). All physical load exposure questions with answer categories (Almost all the time, approximately 3/4 of the time, approximately 1/2 of the time, approximately 1/4 of the time, Rarely, Never) are from the former (1990-2010) Danish work environment cohort study (DWECS) questionnaire (or heavily inspired from it). Hermann Burr was the main researcher involved in the construction of the questions from DWECS, but the Danish labour inspectorate was also involved. All other questions used in the present paper were formulated to the WEHD, and Lars L. Andersen was the main researcher involved. The target population was the Danish working population between 18 and 65 years, employed for a minimum of 35 hours, and with an income of minimum 3000 DKK (approximately 400 €) per month in the last 3 months. In 2014, 50,875 employed workers received an invitation letter to complete a web-based questionnaire (35,023 randomly chosen, 15 852 cohort from 2012). Non-responders received a reminder with a paper version of the questionnaire. Finally, 29,166 answers were returned. The questionnaire includes 51 main questionnaires on psychologic

and physic work environment and on health outcomes. The results from the survey are used in the improvement of the work environment at Danish work places.

Many of the physical workload questions referred to in the present study have proven their predictive validity in epidemiological studies [9-14]. However, if the self-report exposures are compared to objective measurements the accuracy is less convincing [15].

Finland: The Finnish National Work and Health Survey

has been conducted every three years by the Finnish Institute of Occupational Health since 1997. The survey serves as a national surveillance system on perceived working conditions (physical, chemical, ergonomic and psychosocial) and the health of the working-age population.

An expert group of Finnish Institute of Occupational health has planned the content of the interview. The questions concern the following topics: socioeconomic and workplace related background factors, working conditions (physical, ergonomic, chemical, psychosocial) and safety at work, perceived physical and mental strain, organizational culture, gender and age equality, health promotion at the workplace, use of health services, contents of occupational health services, perceived health and work ability, mental well-being, vocational skills, and health related behaviour.

The data was collected through computer-assisted telephone interviews. The random sample from the population register represents the Finnish working age population from 25 to 64 years of age (in 2009 from 20 to 64 and in 2012 from 20 to 68 years of age). In 1997 71%, in 2000 58%, in 2003 67%, in 2006 63%, 2009 59% and 2012 40% of the original sample (currently working) were interviewed. The total number of interviewees is 14,684 currently working Finns, around 2500 interviews in every three year. The sample contain both employees and self-employed persons. The respondents are representative of the Finnish working-age population with reference to sex, age, occupation and socioeconomic status (based on occupation and occupational status), and region. A report and conference papers based on the survey enclosed [16-20].

Netherlands Working Conditions Survey (NWCS)

is a large-scale periodical survey on the working conditions of Dutch employees. Issues addressed in the questionnaire vary from physical and psychological workload, work place bullying to occupational accidents, working hours and development. The data is enriched by Statistics Netherlands with data on earnings, occupation, and demographics. Twelve surveys have been performed to date, in 2005-2013 some 23,000 and in 2014-2016, about 40,000 employees per year have responded to the surveys. The aim of the NWCS is to investigate the quality of work and employment in the Netherlands. A large and representative random sample of employees is taken yearly. The NWCS tracks trends in work risks, the effects of those risks and the measures taken by employers. TNO provides employers' organizations, unions and the Ministry of Social affairs and Employment with national reference data for the purpose of occupational health and safety covenants. The NWCS data is also used to analyse the relationship between work and health, amongst others by linking hospital admission and mortality registers to the survey at employee level in order to study causal relations. Several Dutch papers based on the survey addressing mechanical exposure and health outcomes have been published [21-31].

German BIBB/BAuA Employment Survey 2011/2012

is a survey of the Federal Institute for Vocational Education and Training (BIBB) and the Federal Institute for Occupational Safety and Health (BAuA). It is carried out every sixth year; the last time was in 2011/2012. The survey was first launched in 1979 as a cooperation

of the BIBB and the Institute for Employment Research (IAB). The BAuA became project partner in 1998/1999, and the survey 2005/2006 was jointly implemented by BIBB and BAuA. The topics in the interview are the qualification of the labour force, working and employment conditions as well as job satisfaction and health complaints. The interviews contain constant questions (core variables) to allow for an assessment of status and trends as well as items focusing on specific themes that may change over time. Among other aspects, the survey also covers biomechanical exposures. The questionnaire was designed by researches from BIBB and BAuA. Developing the questionnaire included the exchange with national and international experts in the fields of the survey content issues as well as survey methods. The items of BIBB/BAuA are not protected by copyright, which means any use is possible without informing BAuA. Furthermore, a part of the items was used in the BAuA Working Time Survey 2015, a national survey in Germany on working time issues.
In 2005/2006, the study's mode of data collection changed from computer assisted personal interviews to computer assisted telephone interviews, conducted by professional interviewers from TNS Infratest Sozialforschung (now Kantar public). The study population comprises all people, aged 15 years and over, working at least 10 hours per week. In the 2012 survey, the gross sample was 122,257 subjects and the number of subjects interviewed was 20,036. BAuA publishes fact sheets on specific aspects of the survey, which are all available at https://www.baua.de/DE/Themen/Arbeitswelt-und-Arbeitsschutz-im-Wandel/Arbeitsweltberichterstattung/Arbeitsbedingungen/BIBB-BAuA-2012.html. Anonymized data files are available for researchers not involved in the project through the Research Data Center of the BIBB (https://www.bibb.de/en/15182.php). We enclose a selection of papers published based on BIBB-BAuA referring to physical workloads [32-42]

The Spanish National Working Conditions survey

In order to improve the comparability of Spanish working conditions with those of other EU countries, it was decided to integrate the Spanish and European surveys drawn up by Eurofound, expanding the sample size in Spain to 3362 subjects interviewed. Soon the first edition entitled "6th EWCS - Spain 2015" will be published.

European working conditions survey/Spanish National Working Conditions survey

Since 1991, Eurofound has been monitoring working conditions in Europe through its European Working Conditions Survey (EWCS). The survey aims to measure working conditions across European countries (status and trends), analyse the relationships between different aspects of these, identify groups at risk, highlight issues of concern and areas of progress and, ultimately, contribute to developing EU policy aimed at improving job quality. In 2015, the sixth EWCS interviewed almost 44,000 workers (both employees and self-employed people) in 35 European countries: the 28 EU Member States, the five EU candidate countries, and Norway and Switzerland. Workers were asked a range of questions concerning employment status, work organisation, learning and training, working time duration and organisation, physical and psychosocial risk factors, health and safety, work–life balance, worker participation, earnings and financial security, as well as work and health. A report from the 2015 survey has been published [43]. A former report have compared the European national working conditions surveys [44].

References

1. Aagestad C: **Arbeidsmiljøet i Norge og EU—en sammenlikning (Work envirinment in Norway and EU—a comparison)** *Stami –rapport.* vol. 13. Oslo: National Institute of Occupational Health; 2012.
2. Sterud T: **Work-related psychosocial and mechanical risk factors for work disability: a 3-year follow-up study of the general working population in Norway**. *Scand J Work Environ Health* 2013, **39**(5):468-476.
3. Sterud T: **Work-related mechanical risk factors for long-term sick leave: a prospective study of the general working population in Norway**. *Eur J Public Health* 2014, **24**(1):111-116.
4. Sterud T: **Work-related gender differences in physician-certified sick leave: a prospective study of the general working population in Norway**. *Scand J Work Environ Health* 2014, **40**(4):361-369.
5. Sterud T, Johannessen HA: **Do work-related mechanical and psychosocial factors contribute to the social gradient in long-term sick leave: a prospective study of the general working population in Norway**. *Scand J Public Health* 2014, **42**(3):329-334.
6. Sterud T, Johannessen HA, Tynes T: **Work-related psychosocial and mechanical risk factors for neck/shoulder pain: a 3-year follow-up study of the general working population in Norway**. *Int Arch Occup Environ Health* 2014, **87**(5):471-481.
7. Sterud T, Johannessen HA, Tynes T: **Do Work-Related Mechanical and Psychosocial Factors Contribute to the Social Gradient in Low Back Pain?: A 3-Year Follow-Up Study of the General Working Population in Norway**. *Spine* 2016, **41**(13):1089-1095.
8. Sterud T, Tynes T: **Work-related psychosocial and mechanical risk factors for low back pain: a 3-year follow-up study of the general working population in Norway**. *Occup Environ Med* 2013, **70**(5):296-302.
9. Andersen LL, Fallentin N, Thorsen SV, Holtermann A: **Physical workload and risk of long-term sickness absence in the general working population and among blue-collar workers: prospective cohort study with register follow-up**. *Occup Environ Med* 2016, **73**(4):246-253.
10. Bang Christensen K, Lund T, Labriola M, Villadsen E, Bultmann U: **The fraction of long-term sickness absence attributable to work environmental factors: prospective results from the Danish Work Environment Cohort Study**. *Occup Environ Med* 2007, **64**(7):487-489.
11. Burr H, Pedersen J, Hansen JV: **Work environment as predictor of long-term sickness absence: linkage of self-reported DWECS data with the DREAM register**. *Scand J Public Health* 2011, **39**(7 Suppl):147-152.
12. Moller SV, Hannerz H, Hansen AM, Burr H, Holtermann A: **Multi-wave cohort study of sedentary work and risk of ischemic heart disease**. *Scand J Work Environ Health* 2016, **42**(1):43-51.
13. Thorsen SV, Burr H, Diderichsen F, Bjorner JB: **A one-item workability measure mediates work demands, individual resources and health in the prediction of sickness absence**. *Int Arch Occup Environ Health* 2013, **86**(7):755-766.
14. van der Ploeg HP, Moller SV, Hannerz H, van der Beek AJ, Holtermann A: **Temporal changes in occupational sitting time in the Danish workforce and associations with all-cause mortality: results from the Danish work environment cohort study**. *Int J Behav Nutr Phys Ac* 2015, **12**:71.
15. Gupta N, Heiden M, Mathiassen SE, Holtermann A: **Prediction of objectively measured physical activity and sedentariness among blue-collar workers using survey questionnaires**. *Scand J Work Environ Health* 2016, **42**(3):237-245.
16. Kauppinen T MHP, Perkiö–Mäkelä M, Saalo A, Toikkanen J, Tuomivaara S, Uuksulainen S, Viluksela M, Virtanen S. (ed.). : **Work and Health in Finland 2012**. In. Helsinki: . Finnish Institute of Occupational Healt; 2013.

17. Perkiö–Mäkelä M HM: **Physical work load and thoughts of retirement**. In: *18th World Congress on Ergonomics.* Recife, Brazil; 2012.
18. **Finnish National Work and Health Survey 2012.** [http://www.ttl.fi/tyojaterveys]
19. Perkiö–Mäkelä M KR: **Physical work load in Finland based on the Finnish National Work and Health Survey**. In: *Nordic Ergonomics Society (NES) 40 th Annual Congress Ergonomics is a lifestyle* Reykjavik, Iceland.; 2008.
20. Perkiö–Mäkelä M KR: **Physical work load in Finland based on the Finnish National Work and Health Survey**. In: *Ergonomics is a lifestyle.* vol. 40 th Annual Congress. Reykjavik: Nordic Ergonomics Society (NES); 2008.
21. Akkermans J, Brenninkmeijer V, van den Bossche SN, Blonk RW, Schaufeli WB: **Young and going strong? A longitudinal study on occupational health among young employees of different educational levels**. *Career Dev Int* 2013, **18**(4):416-435.
22. Bekkers MB, Koppes LL, Rodenburg W, van Steeg H, Proper KI: **Relationship of night and shift work with weight change and lifestyle behaviors**. *J Occup Env Med* 2015, **57**(4):e37-44.
23. Boot CR, van den Heuvel SG, Bultmann U, de Boer AG, Koppes LL, van der Beek AJ: **Work adjustments in a representative sample of employees with a chronic disease in the Netherlands**. *J Occup Rehabil* 2013, **23**(2):200-208.
24. de Vries J: **Lichamelijke belasting op het werk en ziekteverzuim**. 2010.
25. de Vroome EM, Uegaki K, van der Ploeg CP, Treutlein DB, Steenbeek R, de Weerd M, van den Bossche SN: **Burden of Sickness Absence Due to Chronic Disease in the Dutch Workforce from 2007 to 2011**. *J Occup Rehabil* 2015, **25**(4):675-684.
26. Fransson EI, Nyberg ST, Heikkila K, Alfredsson L, Bjorner JB, Borritz M, Burr H, Dragano N, Geuskens GA, Goldberg M *et al*: **Job strain and the risk of stroke: an individual-participant data meta-analysis**. *Stroke* 2015, **46**(2):557-559.
27. Oude Hengel KM, Blatter BM, Geuskens GA, Koppes LL, Bongers PM: **Factors associated with the ability and willingness to continue working until the age of 65 in construction workers**. *Int Arch Occup Environ Health* 2012, **85**(7):783-790.
28. Smulders P: **Technologische innovatie naar beroep en werkplek**. *ESB Economisch Statistische Berichten 2015;100(4708):237-239* 2015, **100**(4708):237-239.
29. **Monitoring work & Employment by TNO.**[http://www.monitorarbeid.tno.nl/english/english]
30. van den Bossche S, Souren M: **Werknemers met fysiek ongemakkelijk werk doorgaans minder tevreden**. *TSG* 2011, **89**(2):92-93.
31. Viester L, Verhagen EA, Oude Hengel KM, Koppes LL, van der Beek AJ, Bongers PM: **The relation between body mass index and musculoskeletal symptoms in the working population**. *BMC musculoskelet Disord* 2013, **14**:238.
32. **Hart im Nehmen? - Körperlich harte Arbeit nach wie vor aktuell.** *Faktenblatt 02* [https://www.baua.de/DE/Angebote/Publikationen/Fakten/BIBB-BAuA-02.html]
33. BIBB/BAuA: **Demografischer Wandel in der Arbeit - Körperlich schwere Arbeit belastet Ältere stärker**. *Faktenblatt 05.* [https://www.baua.de/DE/Angebote/Publikationen/Fakten/BIBB-BAuA-05.html]
34. BIBB/BAuA:**Arbeit in der Pflege - Arbeit am Limit? Arbeitsbedingungen in der Pflegebranche.** *Faktenblatt 10.* [https://www.baua.de/DE/Angebote/Publikationen/Fakten/BIBB-BAuA-10.html]
35. BIBB/BAuA:**Arbeitsbedingungen am Bau - Immer noch schwere körperliche Arbeit trotz technischen Fortschritts.** *Faktenblatt 11.* [https://www.baua.de/DE/Angebote/Publikationen/Fakten/BIBB-BAuA-11.html]
36. BIBB/BAuA:**Arbeitsbedingungen in der deutschen Automobilindustrie.** *Faktenblatt 13.* [https://www.baua.de/DE/Angebote/Publikationen/Fakten/BIBB-BAuA-13.html]
37. Gensicke MT & Tschersicht N: **Methodenexperiment im Rahmen der BIBB/BAuA-Erwerbstätigenbefragung 2011/2012: Ein Vergleich von CATI, CAPI und CAWI**. In. Dortmund: Bundesanstalt für Arbeitsschutz und Arbeitsmedizin 2014.

38. Hall AS & Siefer A: **Fragebogen der BIBB/BAuA-Erwerbstätigenbefragung 2012**. In. Bonn & Dortmund: Bundesinstitut für Berufsbildung & Bundesanstalt für Arbeitsschutz und Arbeitsmedizin; 2012.
39. Latza U, Liebers F, Franke F: **Körperliche Beanspruchung und physische Belastungen bei der Arbeit–ein abnehmendes Public Health-Problem?** In: *Public Health Forum: 2013*: Elsevier; 2013: 12. e11-12. e13.
40. Liebers F, Brendler, C. & Latza, U: **Risikogruppen für Muskel-Skelett- und Herz-Kreislauf-Erkrankungen**. In: *Sicherheit und Gesundheit bei der Arbeit 2013 Unfallverhütungsbericht Arbeit (Suga).* edn. Edited by BMAS/BAuA. Dortmund/Berlin: Dresden: Bundesministerium für Arbeit und Soziales (BMAS)2014: 108-118.
41. Lohmann-Haislah A: **Stressreport Deutschland 2012. Psychische Anforderungen, Ressourcen und Befinden**. In. Dortmund: Bundesanstalt für Arbeitsschutz und Arbeitsmedizin; 2012.
42. Wittig P, Nöllenheidt, C. & Brenscheidt, S.: **Grundauswertung der BIBB/BAuA-Erwerbstätigenbefragung 2012** Dortmund: Bundesanstalt für Arbeitsschutz und Arbeitsmedizin 2013.
43. Parent-Thirion A, Vermeylen G, van Houten G, Lyly-Yrjänäinen M, Biletta I, Cabrita J: **Sixth European Working Conditions Survey-Overview Report**. In: *Publications Office of the European Union.* Luxembourg: EUROFOUND; 2016.
44. Cabrita J, & Peycheva, D. : **National working conditions surveys in Europe: A compilation.** *European Foundation for the Improvement of Living and Working Conditions.* Luxembourgh; 2014. [http://digitalcommons.ilr.cornell.edu/cgi/viewcontent.cgi?article=1403&context=intl]
