# Supplementary material for: Physical working conditions as covered in European monitoring questionnaires
Source: BMC Public Health. 2017 Jun 5;17:544. doi: 10.1186/s12889-017-4465-7 (PMC5460526; doi:10.1186/s12889-017-4465-7)
Supplement: Supplementary file 2 — Overview of all dimensions and items assessing physical workloads in the six surveys. (PDF 65 kb) [file 12889_2017_4465_MOESM2_ESM.pdf]

Country		Denmark	Norway	Finland	Netherlands	Germany	Spain	Europe
**Domain**	**Dimesions**	Work Environment and Health in Denmark (survey 2012, 2014, 2016)	Survey of living conditions: Working environment	Finland: The Finnish National Work and Health Survey	Netherlands Working Conditions Survey	German BIBB/BAuA Employment Survey 2011/2012	Spanish National Working Conditions survey	European working conditions survey
**Lifting, holding & carrying of loads /pushing & pulling of loads**	**Lifting, holding & carrying of loads**	How much of your time at work do you carry or lift objects? (Is your answer "never" please skip to question 27)[almost all the time, approx. 3/4 of the time, approx 1/2 of the time, approx 1/4 of the time, rarely,never]	Do have to lift anything that weighs more than 20 kg on a daily basis, and if so how many times a day? [Yes, at least 20 times a day, yes, 5-19 times, yes, 1-4 times,no]	Does your work involve lifting or carrying by hand, without any equipment?<br>[1 not at all<br>If yes<br>2 occasionally<br>3 weekly<br>4 Daily<br>5 several times a day<br>6 or several times an hour ?<br>8 Cannot say<br>9 No answer]		I will now read out a number of working conditions to you. For each item, please tell me whether these occur on your occupation as <insert occupation from F100-102> frequently, sometimes, rarely or never.<br>- Lifting and carrying loads of more than <for male target persons insert: 20 kg, for females insert: 10 kg ><br>[1: frequently; 2: sometimes; 3: rarely; 4: never]		
		How much does what you carry or lift typically weigh? [Less than 5 kg, 5-15 kg 16-29kg,30 kg or more]	Do have to lift anything that weighs more than 10 kg on a daily basis, and if so how many times a day? [Yes, at least 20 times a day, yes, 5-19 times, yes, 1-4 times,no]	Do the loads that you normally handle just manually, weight [less than 5 kilos, 5-25 kilos or over 25 kilos?]			Please tell me, using the same scale, does your main paid job involve lifting or moving people?<br>[1 All of the time,<br>2 Almost all of the time,<br>3 Around ¾ of the time,<br>4 Around half of the time,<br>5 Around ¼ of the time,<br>6 Almost never,<br>7 Never,<br>8 DK (don't know),<br>9 Ref]	Please tell me, using the same scale, does your main paid job involve lifting or movin g people?<br>[1 All of the time,<br>2 Almost all of the time,<br>3 Around ¾ of the time,<br>4 Around half of the time,<br>5 Around ¼ of the time,<br>6 Almost never,<br>7 Never,<br>8 DK (don't know),<br>9 Ref]
		When necessary, how often do you use aids during lifts or moving of things or individuals? [always, often, sometimes, rarely, never]	If yes to the two questions above . are you normally lifting people? [YES/NO]					
	**Pushing and pulling of loads**	How much of your time at work do you push or pull something? [almost all the time, approx. 3/4 of the time, approx 1/2 of the time, approx 1/4 of the time, rarely,never]						
		How often does it happen that you lift or move things or individuals, even though you ought to be two doing it? [always, often ,sometimes ,rarely, never]						
	**Mixture of both**				Does your job require you to apply a lot of force (pushing/lifting/etc.) or do you use equipment or appliances that require you to use a lot of force ?<br>[yes regularly, yes sometimes, no]		… carrying or moving heavy loads?<br>[1 All of the time,<br>2 Almost all of the time,<br>3 Around ¾ of the time,<br>4 Around half of the time,<br>5 Around ¼ of the time,<br>6 Almost never,<br>7 Never,<br>8 DK (don't know),<br>9 Ref]	… carrying or moving heavy loads?<br>[1 All of the time,<br>2 Almost all of the time,<br>3 Around ¾ of the time,<br>4 Around half of the time,<br>5 Around ¼ of the time,<br>6 Almost never,<br>7 Never,<br>8 DK (don't know),<br>9 Ref]
**Manual work processes / repetitive hand-arm movements**	**Repetetive movements**	How much of your time at work do you make the same arm movements several times a minute (for example, packaging , assembly, machine feeding, cutting)?[almo all the time, approx. 3/4 of the time, approx 1/2 of the time, approx 1/4 of the time, rarely,never]	Does your work involve repeated movements with just one of your hands or arms? Yes/no. If yes, how much of the time do you do this during a normal working day' [[almost the whole time, approx. 3/4 of the time, approx. half of the time, approx. 1/4 of the time, very little of the time]	Does your work involve repeated hand movements several times a minute? These occur in such things as production line and assembly work, pricing goods or goods from a conveyor belt. This question does not mean writing on a typewriter or computer.<br>[1 not at all<br>If yes<br>2 daily for at least 1-2 hours<br>3 less than an hour a day<br>4 almost every day<br>5 or occasionally?]	Does your job require you to make repetitive movements?<br>[yes regularly, yes sometimes, no]		Please tell me, using the same scale, does your main paid job involve repetitive hand or arm movements?<br>[1 All of the time,<br>2 Almost all of the time,<br>3 Around ¾ of the time,<br>4 Around half of the time,<br>5 Around ¼ of the time,<br>6 Almost never,<br>7 Never,<br>8 DK (don't know),<br>9 Ref]	Please tell me, using the same scale, does your main paid job involve repetitive hand or arm movements?<br>[1 All of the time,<br>2 Almost all of the time,<br>3 Around ¾ of the time,<br>4 Around half of the time,<br>5 Around ¼ of the time,<br>6 Almost never,<br>7 Never,<br>8 DK (don't know),<br>9 Ref]

Country		Denmark	Norway	Finland	Netherlands	Germany	Spain	Europe
**Domain**	**Dimesions**	Work Environment and Health in Denmark (survey 2012, 2014, 2016)	Survey of living conditions: Working environment	Finland: The Finnish National Work and Health Survey	Netherlands Working Conditions Survey	German BIBB/BAuA Employment Survey 2011/2012	Spanish National Working Conditions survey	European working conditions survey
**Lifting, holding & carrying of loads /pushing & pulling of loads**	**Lifting, holding & carrying of loads**	How much of your time at work do you carry or lift objects? (Is your answer "never" please skip to question 27)[almost all the time, approx. 3/4 of the time, approx 1/2 of the time, approx 1/4 of the time, rarely,never]	Do have to lift anything that weighs more than 20 kg on a daily basis, and if so how many times a day? [Yes, at least 20 times a day, yes, 5-19 times, yes, 1-4 times,no]	Does your work involve lifting or carrying by hand, without any equipment?<br>[1 not at all<br>If yes<br>2 occasionally<br>3 weekly<br>4 Daily<br>5 several times a day<br>6 or several times an hour ?<br>8 Cannot say<br>9 No answer]		I will now read out a number of working conditions to you. For each item, please tell me whether these occur on your occupation as <insert occupation from F100-102> frequently, sometimes, rarely or never.<br>- Lifting and carrying loads of more than <for male target persons insert: 20 kg, for females insert: 10 kg ><br>[1: frequently; 2: sometimes; 3: rarely; 4: never]		
**Working standing / walking**	**Working standing only**		Do you work standing up? [almost the whole time, approx. 3/4 of the time, approx. half of the time, approx. 1/4 of the time, very little of the time]			I will now read out a number of working conditions to you. For each item, please tell me whether these occur on your occupation as <insert occupation from F100-102> frequently, sometimes, rarely or never.<br>- Working while standing.<br>How frequently does this happen?<br>[1: frequently; 2: sometimes; 3: rarely; 4: never]	… standing?<br>[1 All of the time,<br>2 Almost all of the time,<br>3 Around ¾ of the time,<br>4 Around half of the time,<br>5 Around ¼ of the time,<br>6 Almost never,<br>7 Never,<br>8 DK (don't know),<br>9 Ref]	… standing?<br>[1 All of the time,<br>2 Almost all of the time,<br>3 Around ¾ of the time,<br>4 Around half of the time,<br>5 Around ¼ of the time,<br>6 Almost never,<br>7 Never,<br>8 DK (don't know),<br>9 Ref]
	**Working walking only**		How much of the time do you walk around during a normal working day? [almost the whole time, approx. 3/4 of the time, approx. half of the time, approx. 1/4 of the time, very little of the time]					
	**Mixture of standing and walking**	How much of your time at work do you walk or stand? [almost all the time, approx. 3/4 of the time, approx 1/2 of the time, approx 1/4 of the time, rarely,never]		Is the nature of your work mainly<br>[1 sedentary<br>2 or are you standing or walking<br>3 changing betwen walking, standing and sitting]				
**Working sitting**	**Working sitting**	How much of your time at work do you sit down? [almost all the time, approx. 3/4 of the time, approx 1/2 of the time, approx 1/4 of the time, rarely,never]	How much of the time do you work sitting down during a normal working day? almost the whole time, approx. 3/4 of the time, approx. half of the time, approx. 1/4 of the time, very little of the time	Is the nature of your work mainly<br>[1 sedentary<br>2 or are you standing or walking<br>3 changing betwen walking, standing and sitting]				
**Awkward body postures**	**Work at or above shoulder height**	How much of your time at work do you keep your arms lifted at or above shoulder level? [almost all the time, approx. 3/4 of the time, approx 1/2 of the time, approx 1/4 of the time, rarely,never]	Do you work with your hands raised to shoulder height or higher? [almost the whole time, approx. 3/4 of the time, approx. half of the time, approx . 1/4 of the time, very little of the time]	Does your work involve working with one or both hands above shoulder height?<br>[1 not at all<br><br>If yes<br>2 daily for at least 1-2 hours<br>3 less than an hour a day<br>4 almost every day<br>5 or occasionally?]				
	**Working with back in awkward position**	How much of your time at work do you work with your back twisted or doubled over without supporting yourself with your hands or arms?[almost all the time, approx. 3/4 of the time, approx 1/2 of the time, approx 1/4 of the time, rarely,never]	Do you work in positions where you are leaning forward without supporting yourself on your hands or arms? Yes/no. If yes, how much of the time do you do this during a normal working day? [almost the whole time, approx. 3/4 of the time, approx. half of the time, approx. 1/4 of the time, very little of the time] Two follow up questions to this question under taxonomy mixed exposures)	Does your work involve stooping or otherwise having your back in an awkward working position?<br>[1 not at all<br>If yes<br>2 daily for at least 1-2 hours<br>3 less than an hour a day<br>4 almost every day<br>5 or occasionally?]				
			When working in those positions [where you are leaning forward without supporting yourself on your hands or arms], do you work in those positions with your back twisted? [Yes/no]					
			When working like this [where you are leaning forward without supporting yourself on your hands or arm with your back twisted] do you need to lift anything that weighs more than 10 kg? [YES/NO]					

Country		Denmark	Norway	Finland	Netherlands	Germany	Spain	Europe
**Domain**	**Dimesions**	Work Environment and Health in<br>Denmark (survey 2012, 2014, 2016)	Survey of living conditions:<br>Working environment	Finland: The Finnish National<br>Work and Health Survey	Netherlands Working Conditions Survey	German BIBB/BAuA Employment<br>Survey 2011/2012	Spanish National Working<br>Conditions survey	European working conditions survey
**Lifting, holding & carrying of loads /pushing & pulling of loads**	**Lifting, holding & carrying of loads**	How much of your time at work do you<br>carry or lift objects?<br>(Is your answer "never" please skip<br>to question 27)[almost all the time,<br>approx. 3/4 of the time,<br>approx 1/2 of the time, approx<br>1/4 of the time, rarely,never]	Do have to lift anything that weighs more<br>than 20 kg on a daily basis, and if so how<br>many times a day?<br>[Yes, at least 20 times<br>a day, yes, 5-19 times, yes, 1-4 times,no]	Does your work involve lifting<br>or carrying by hand, without<br>any equipment?<br>[1 not at all<br>If yes<br>2 occasionally<br>3 weekly<br>4 Daily<br>5 several times a day<br>6 or several times an hour ?<br>8 Cannot say<br>9 No answer]		I will now read out a number of<br>working conditions to you.<br>For each item, please tell me<br>whether these occur on your<br>occupation as <insert occupation<br>from F100-102> frequently,<br>sometimes, rarely or never.<br>- Lifting and carrying loads<br>of more than <for male target<br>persons insert: 20 kg,<br>for females insert: 10 kg ><br>[1: frequently; 2: sometimes;<br>3: rarely; 4: never]		
	**Working squatting and kneeing**	How much of your time at work do you<br>squat or lie on your knees when you are wor<br>[almost all the time, approx. 3/4 of the time,<br>approx 1/2 of the time, approx 1/4 of the time<br>rarely,never]	Do you need to squat or kneel in the<br>course of your work? [almost the whole<br>time, approx. 3/4 of the time,<br>approx. half of the time, approx.<br>1/4 of the time, very little of the time]					
	**Working with head bent forward**		Do you work with your head bent<br>forward? Yes/no. If yes, how much<br>of the time do you do this during a normal<br>working da? [almost the whole time,<br>approx. 3/4 of the time, approx.<br>half of the time, approx.<br>1/4 of the time, very little of the time]					
	**Working in specified awkward body postures**					- Working in a bent over, squatting,<br>kneeing or lying position, working above<br>your head [1: frequently;<br>2: sometimes; 3: rarely; 4: never]		
	**Working in uncomfortable and tiring body postures**				Do you have to work in uncomfortable<br>body postures [yes regularly,<br>yes sometimes, no]?		Please tell me, using the same scale,<br>your main paid job involve tiring<br>or painful positions?<br>[1 All of the time,<br>2 Almost all of the time,<br>3 Around ¾ of the time,<br>4 Around half of the time,<br>5 Around ¼ of the time,<br>6 Almost never,<br>7 Never,<br>8 DK (don't know),<br>9 Ref]	Please tell me, using the same scale,<br>your main paid job involve tiring<br>or painful positions?<br>[1 All of the time,<br>2 Almost all of the time,<br>3 Around ¾ of the time,<br>4 Around half of the time,<br>5 Around ¼ of the time,<br>6 Almost never,<br>7 Never,<br>8 DK (don't know),<br>9 Ref]
**Physical work effort**	**Strenuous work**	How physical strenuous do you normally per<br>your current work to be?<br>[0-not at all, 1,2,3,4,5,6,7,8,9,10<br>-maximum hard]	Does your work involve so much effort<br>that it causes you to breathe more rapidly?<br>[almost the whole time,<br>approx. 3/4 of the time,<br>approx. half of the time,<br>approx. 1/4 of the time,<br>very little of the time]	Is your work physically light, fairly light,<br>[a bit strenuous,<br>quite strenuous or<br>very strenuous ?]				
	**Strenuous lifting**	How often do you find the typical lifts at<br>work strenuous?<br>[always, often,sometimes,rarely,never]						
**Vibrations**	**Vibrations unspecified**	How much of your time at work are<br>you subjected to intense vibrations<br>(for example from a tractor, truck etc.)?<br>[almost all the time,<br>approx. 3/4 of the time,<br>approx 1/2 of the time,<br>approx 1/4 of the time, rarely,never]		Is there vibration in your<br>working environment or in your work?<br>[1There isn't any<br>If there is,<br>Does it bother you<br>2 not at all<br>3 a little<br>4 quite a lot<br>5 or a lot<br>8 Cannot say<br>9 No answer]		I will now read out a number of working<br>conditions to you. For each item,<br>please tell me whether these occur on<br>your occupation as <insert occupation<br>from F100-102> frequently, sometimes,<br>rarely or never.<br>- Working with heavy vibrations<br>and reverberations that you can feel<br>in your body<br>[1: frequently; 2: sometimes;<br>3: rarely; 4: never]	Please tell me, using the<br>following scale, are you<br>exposed at work to …<br>vibrations from hand tools, machinery, etc?<br>[1 All of the time,<br>2 Almost all of the time,<br>3 Around ¾ of the time,<br>4 Around half of the time,<br>5 Around ¼ of the time,<br>6 Almost never,<br>7 Never,<br>8 DK (don't know),<br>9 Ref]	Please tell me, using the<br>following scale, are you<br>exposed at work to …<br>vibrations from hand tools, machinery, etc?<br>[1 All of the time,<br>2 Almost all of the time,<br>3 Around ¾ of the time,<br>4 Around half of the time,<br>5 Around ¼ of the time,<br>6 Almost never,<br>7 Never,<br>8 DK (don't know),<br>9 Ref]
	**Arm/hand vibrations**		Are you, in your day-to-day work,<br>exposed to vibrations from machines<br>or tools that you hold in your hands?<br>[almost the whole time,<br>approx. 3/4 of the time,a<br>pprox. half of the time,approx.<br>1/4 of the time,very little of the time]		Does your job require you to<br>use tools that shake or<br>tremble heavily?<br>[yes regularly, yes sometimes, no]			

Country		Denmark	Norway	Finland	Netherlands	Germany	Spain	Europe
**Domain**	**Dimesions**	Work Environment and Health in Denmark (survey 2012, 2014, 2016)	Survey of living conditions: Working environment	Finland: The Finnish National Work and Health Survey	Netherlands Working Conditions Survey	German BIBB/BAuA Employment Survey 2011/2012	Spanish National Working Conditions survey	European working conditions survey
**Lifting, holding & carrying of loads /pushing & pulling of loads**	**Lifting, holding & carrying of loads**	How much of your time at work do you carry or lift objects? (Is your answer "never" please skip to question 27)[almost all the time, approx. 3/4 of the time, approx 1/2 of the time, approx 1/4 of the time, rarely,never]	Do have to lift anything that weighs more than 20 kg on a daily basis, and if so how many times a day? [Yes, at least 20 times a day, yes, 5-19 times, yes, 1-4 times,no]	Does your work involve lifting or carrying by hand, without any equipment?<br>[1 not at all<br>If yes<br>2 occasionally<br>3 weekly<br>4 Daily<br>5 several times a day<br>6 or several times an hour ?<br>8 Cannot say<br>9 No answer]		I will now read out a number of working conditions to you. For each item, please tell me whether these occur on your occupation as <insert occupation from F100-102> frequently, sometimes, rarely or never.<br>- Lifting and carrying loads of more than <for male target persons insert: 20 kg, for females insert: 10 kg ><br>[1: frequently; 2: sometimes; 3: rarely; 4: never]		
	**Whole body vibrations**		Are you, in your day-to-day work, exposed to vibrations that cause your whole body to shake, e.g. from a tractor, forklift truck or other piece of machinery?<br>[almost the whole time,<br>approx. 3/4 of the time,<br>approx. half of the time,<br>approx. 1/4 of the time,<br>very litte of the time]					
**Work with computer**	**Work with computers/laptops/smartphone**		Do you work in front of a computer screen?	Do you use a personal computer or computer terminal regularly at work [no/yes]		Please think about your job as <insert occupation from F100-102>. I am now going to name some selected activities. Please tell me how often these activities occur during your work: frequently, sometimes or never.<br>- Working with computers<br>[1: frequently; 2: sometimes; 3: never]	Please tell me, using the same scale, does your main paid job involve working with computers, laptops, smartphones etc.<br>[1 All of the time,<br>2 Almost all of the time,<br>3 Around ¾ of the time,<br>4 Around half of the time,<br>5 Around ¼ of the time,<br>6 Almost never,<br>7 Never,<br>8 DK (don't know),<br>9 Ref]	Please tell me, using the same scale, does your main paid job involve working with computers, laptops, smartphones etc.<br>[1 All of the time,<br>2 Almost all of the time,<br>3 Around ¾ of the time,<br>4 Around half of the time,<br>5 Around ¼ of the time,<br>6 Almost never,<br>7 Never,<br>8 DK (don't know),<br>9 Ref]
			Do you work with a keyboard or mouse? yes/no	Is it a laptop computer? [no/yes]		-Using the Internet or processing emails<br>[1: frequently; 2: sometimes; 3: never]		
			How many hours do you spend doing so on a normal working day? Include work that you do at home, but not personal use. ROUND TO THE NEAREST WHOLE HOUR. HOURS [0..24]	Does your workplace have a separate keyboard and screen for your laptop computer? [no/yes]		What percentage of your working hours in your occupation as <insert occupation from F100-102> do you spend on average working with computers?		
			How many hours do you spend doing so on a normal working day? Include work that you do at home, but not personal use.<br>ROUND TO THE NEAREST WHOLE HOUR. HOURS [0..24]	Do you use a personal computer, computer terminal or laptop computer<br>[1 for less than an hour<br>2 1-2 hours<br>3 3-4 hours<br>4 over 4 hours<br>5 almost every day<br>6 or more rarely?]				
**General / mixed exposure**	**Strong gripping and turning movements of the hand**			Does your work involve strong gripping or turning movements of your hand such as using a screwdriver, cutting, twisting or moving heavy objects manually ?<br>[1 not at all<br>If yes<br>2 daily for at least 1-2 hours<br>3 less than an hour a day<br>4 almost every day<br>5 or occasionally?]				
	**Manual work that requires great dexterity, fast sequences of movements or greater strength**					I will now read out a number of working conditions to you. For each item, please tell me whether these occur on your occupation as <insert occupation from F100-102> frequently, sometimes, rarely or never.<br>- Doing manual work that requires great dexterity, fast sequences of movements or greater strength<br>[1: frequently; 2: sometimes; 3: rarely; 4: never]		
